# Supplementary material for: Identification of Hub Genes Correlated with the Initiation and Progression of CKD in the Unilateral Ureteral Obstruction Model
Source: Biomedicines. 2025 May 27;13(6):1316. doi: 10.3390/biomedicines13061316 (PMC12189713; doi:10.3390/biomedicines13061316)
Supplement: Supplementary file 1 [file biomedicines-13-01316-s001.zip › biomedicines-3602478-supplementary.pdf]

Supplementary Materials:

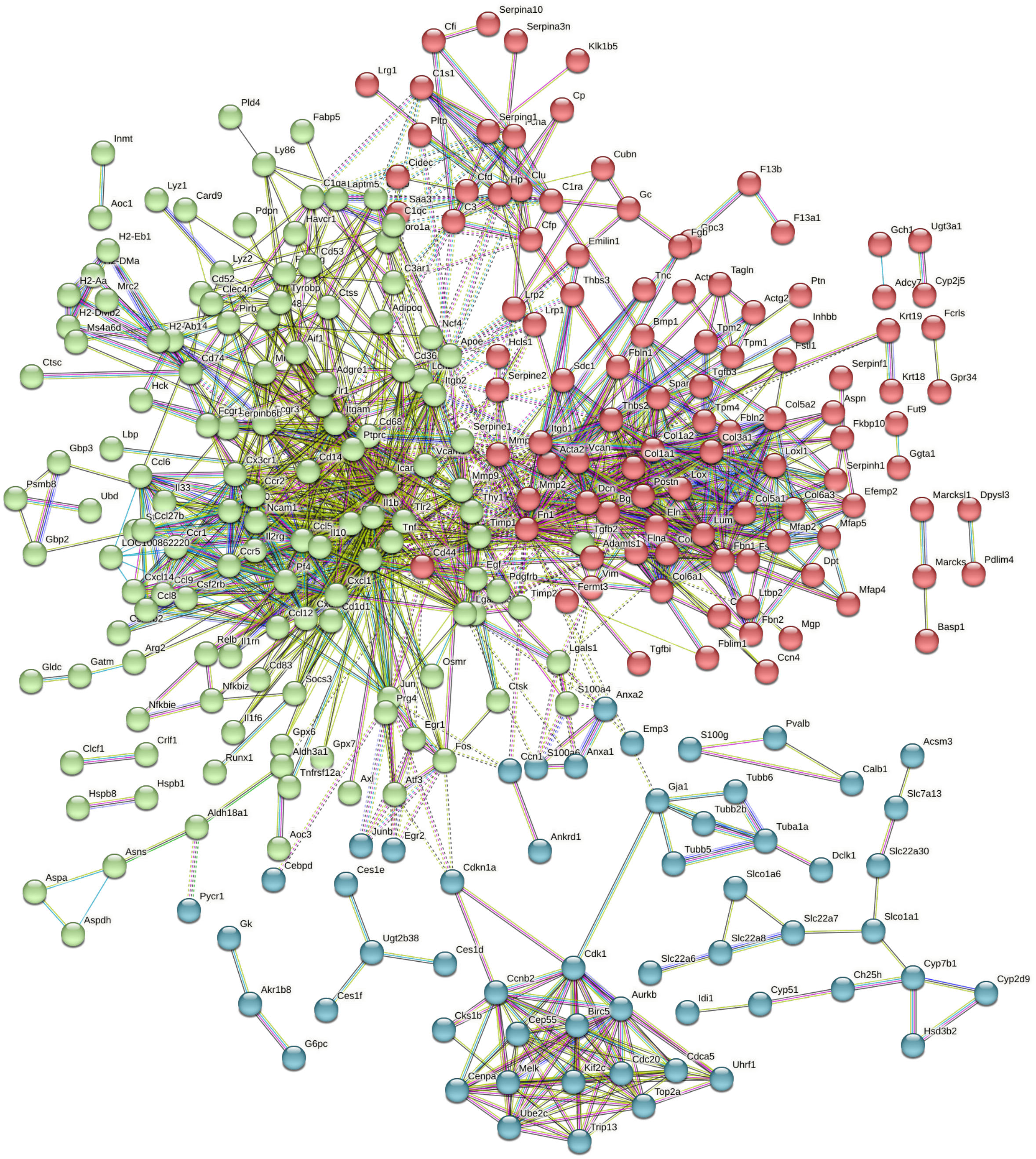

Supplementary Figure S1. PPI network analysis of DEGs. PPI network analysis using online database STRING software. The nodes in cluster represented different proteins, and the edges between the nodes indicated interactions between two proteins.

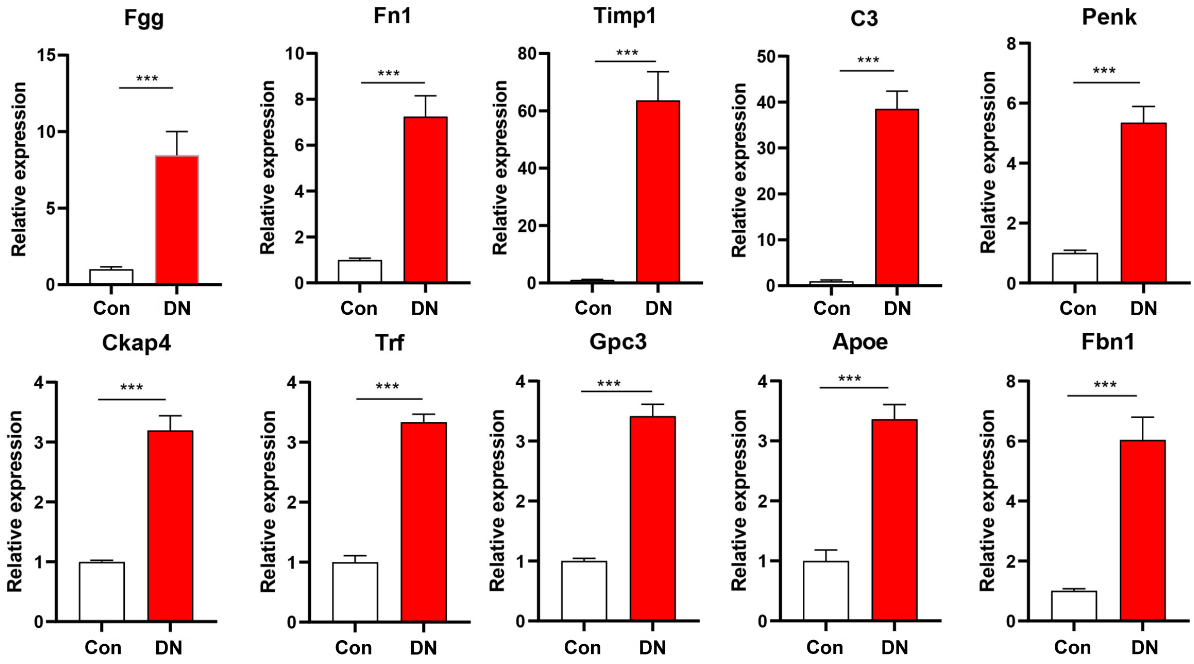

Supplementary Figure S2. The hub gene mRNA expression in GSE30528 database. The mRNA levels of hub genes in GSE30528 are presented. ** $p < 0.01$, *** $p < 0.001$.
